# Effects of vitamin D_2_ or D_3_ supplementation on glycaemic control and cardiometabolic risk among people at risk of type 2 diabetes: results of a randomized double‐blind placebo‐controlled trial

**DOI:** 10.1111/dom.12625

**Published:** 2016-02-04

**Authors:** N. G. Forouhi, R. K. Menon, S. J. Sharp, N. Mannan, P. M. Timms, A. R. Martineau, A. P. Rickard, B. J. Boucher, T. A. Chowdhury, C. J. Griffiths, S. E. Greenwald, S. J. Griffin, G. A. Hitman

**Affiliations:** ^1^ Medical Research Council Epidemiology UnitUniversity of Cambridge School of Clinical MedicineCambridgeUK; ^2^ Blizard InstituteQueen Mary University of LondonLondonUK; ^3^ Homerton University Hospital NHS Foundation TrustLondonUK; ^4^ Barts Healthcare NHS TrustLondonUK

**Keywords:** intervention, placebo, pulse wave velocity, randomized, trial, type 2 diabetes, vitamin D_2_, vitamin D_3_

## Abstract

**Aims:**

To investigate the effect of short‐term vitamin D supplementation on cardiometabolic outcomes among individuals with an elevated risk of diabetes.

**Methods:**

In a double‐blind placebo‐controlled randomized trial, 340 adults who had an elevated risk of type 2 diabetes (non‐diabetic hyperglycaemia or positive diabetes risk score) were randomized to either placebo, 100 000 IU vitamin D_2_ (ergocalciferol) or 100 000 IU vitamin D_3_ (cholecalciferol), orally administered monthly for 4 months. The primary outcome was change in glycated haemoglobin (HbA1c) between baseline and 4 months, adjusted for baseline. Secondary outcomes included: blood pressure; lipid levels; apolipoprotein levels; C‐reactive protein levels; pulse wave velocity (PWV); anthropometric measures; and safety of the supplementation.

**Results:**

The mean [standard deviation (s.d.)] 25‐hydroxyvitamin D [25(OH)D]_2_ concentration increased from 5.2 (4.1) to 53.9 (18.5) nmol/l in the D_2_ group, and the mean (s.d.) 25(OH)D_3_ concentration increased from 45.8 (22.6) to 83.8 (22.7) nmol/l in the D_3_ group. There was no effect of vitamin D supplementation on HbA1c: D_2_ versus placebo: −0.05% [95% confidence interval (CI) −0.11, 0.02] or −0.51 mmol/mol (95% CI −1.16, 0.14; p = 0.13); D_3_ versus placebo: 0.02% (95% CI −0.04, 0.08) or 0.19 mmol/mol (95% CI −0.46, 0.83; p = 0.57). There were no clinically meaningful effects on secondary outcomes, except PWV [D_2_ versus placebo: −0.68 m/s (95% CI −1.31, −0.05); D_3_ versus placebo −0.73 m/s (95% CI −1.42, −0.03)]. No important safety issues were identified.

**Conclusions:**

Short‐term supplementation with vitamin D_2_ or D_3_ had no effect on HbA1c. The modest reduction in PWV with both D_2_ and D_3_ relative to placebo suggests that vitamin D supplementation has a beneficial effect on arterial stiffness.

## Introduction

There is epidemiological evidence for an inverse association between circulating 25‐hydroxyvitamin D [25(OH)D] concentration, an indicator of vitamin D status, and risk of type 2 diabetes; however, overall evidence from clinical trials does not support a causal association for diabetes incidence 1 or intermediate metabolic markers 2, 3, although some clinical trials have reported mixed findings 4, 5. Evidence for an inverse association between circulating 25(OH)D concentrations and cardiovascular outcomes has also been accumulating 6, but trial evidence does not clearly support a causal effect for incident events or intermediate markers such as blood pressure, lipid levels, inflammation and arterial stiffness 6, 7, 8, 9, 10, 11, 12.

Taken together, the current evidence remains inconclusive, yet there is continued interest in the potential role of vitamin D in cardiometabolic risk protection. Concurrently, while many would consider vitamin D_3_ (cholecalciferol) more effective than vitamin D_2_ (ergocalciferol) in maintaining circulating concentrations of 25(OH)D 13, 14, there is a separate debate regarding bioavailability, with a higher proportion of ‘free’ (i.e. unbound) or bioavailable 25(OH)D_2_ than free/bioavailable 25(OH)D_3_ in response to an equivalent oral dose.

We conducted a trial to determine whether short‐term oral supplementation given monthly with vitamin D_2_ or vitamin D_3_ can lead to a reduction in glycaemia and an improvement in cardiometabolic factors in people at risk of developing type 2 diabetes. We also examined the feasibility and safety of relatively high dose vitamin D supplementation among individuals we drew from the general population without prior knowledge of their circulating 25(OH)D concentrations.

## Methods

### Trial Design and Participants

We designed a double‐blind, placebo‐controlled randomized clinical trial in people at risk of developing type 2 diabetes, across two sites: East London and Cambridge, UK 15. We randomly allocated 340 participants to one of three groups who received a monthly oral dose for 4 months of 100 000 IU (equivalent to 2.5 mg) of either ergocalciferol (vitamin D_2_) or cholecalciferol (vitamin D_3_) or a monthly oral dose of placebo. Each participant was followed up for a total of 4 months from their first visit. The first three doses were given at the clinic visit, while the final dose was taken at home after contact by the study team, and their investigational medicinal products (IMPs) consumption status was recorded (complete, partial or failed). The two IMPs included ergocalciferol (Sterogyl, containing 20 000 IU vitamin D_2_ per ml in ethanol) or cholecalciferol (Vigantol oil, containing 20 000 IU vitamin D_3_ per ml in Miglyol^®^ vehicle oil), representing a daily dose equivalent of ∼3300 IU. The placebo was Miglyol oil with esters of coconut and palm‐derived oils.

Details of the exclusion and inclusion criteria have been described previously 15. Men and women aged 30–75 years registered with a general practitioner (London) or already part of an ongoing observational study that was drawn from lists of general practitioners (Cambridge), from any ethnic group were eligible if they had an elevated risk of developing type 2 diabetes, with either: (i) the presence of non‐diabetic hyperglycaemia defined by either impaired glucose tolerance or impaired fasting glucose (World Health Organization criteria), or glycated haemoglobin (HbA1c) levels of 5.5–6.49% (37–47 mmol/mol) or (ii) the presence of Cambridge Risk Score thresholds that indicate elevated risk of diabetes 16. Ethical approval for the trial was provided by the relevant ethics committees and written informed consent was obtained from all participants.

The trial was registered under the numbers: EudraCT 2009‐011264‐11; ISRCTN86515510.

### Trial Outcomes

The primary efficacy outcome of the trial was a change in the HbA1c concentration. There were multiple secondary cardiometabolic outcomes, including systolic and diastolic blood pressure, random cholesterol, HDL cholesterol, apolipoprotein (Apo)A1 and ApoB, cardiovascular disease (CVD) risk score, as assessed by the UK Prospective Diabetes Study (UKPDS) risk engine (version 2) 17 and additionally in London only, a measurement of arterial stiffness assessed by pulse wave velocity (PWV). Further secondary outcomes (both sites) included anthropometry and serum concentrations of high‐sensitivity C‐reactive protein (hsCRP), fructosamine and parathyroid hormone (PTH). Other outcomes included the safety of oral vitamin D without a pre‐assessment of vitamin D status and the feasibility and safety of the intervention 15. For safety, for all recruited participants at each trial visit, we recorded point‐of‐care ionized calcium, laboratory serum‐corrected calcium and laboratory urinary calcium to creatinine ratio. Trial participants who had an elevated point‐of‐care ionized calcium level (>1.3 mmol/l), an elevated urine calcium:creatinine ratio (molar ratio >1) or an elevated serum corrected calcium level (>2.65 mmol/l) were excluded from further doses of the IMP, but continued to be followed up. Provision was made for the recording of adverse events or reactions.

### Clinical and Laboratory Measurements

Systolic and diastolic blood pressure, weight, height and waist circumference were measured according to standardized protocols. Body mass index was calculated as weight (kg) divided by the square of height (m). PWV as a marker of arterial stiffness was measured by a single operator detecting the flow pulses in the carotid and femoral arteries in accordance with recommended procedures 18 using handheld ultrasonic Doppler flow velocimeters (Dopplex MDII, Huntleigh Healthcare, Cardiff, UK) driving pencil probes (4 and 8 MHz) placed over the carotid and femoral arteries, respectively. The output from the velocimeters was passed to a custom‐built data acquisition system 19, sampling at a rate of 1 kHz, linked to a computer which displayed the maximum velocity signals from the two sites in real time. The mean PWV for at least 10–30 s of data, free of movement artifacts, was recorded for each measurement session.

Baseline non‐fasted blood samples were collected from all participants during the first visit, to assess concentrations of serum ionized calcium as well as serum 25(OH)D vitamin D, HbA1c and other secondary biochemical endpoints. During the second and third visits, blood samples were collected only for safety analysis, and during the final (fourth) visit all blood tests were repeated, as during the first visit. HbA1c samples from the first and fourth visit were analysed immediately, while aliquots for all other assays were stored frozen at −70 °C and measured at the end of the trial.

We measured HbA1c levels according to International Federation of Clinical Chemistry and Laboratory Medicine standards in both trial centres and reported them additionally in Diabetes Control and Complications Trial units. Serum 25(OH)D_2_ and D_3_ were measured using the liquid chromatography/tandem mass spectrometry method, with participation in the Vitamin **D E**xternal **Q**uality **A**ssessment **S**cheme (DEQAS) quality assurance scheme.

### Sample Size

Sample size calculations estimated that 207 participants (69 per randomized group) were required to detect a 2.19 mmol/mol (0.2%) difference in mean HbA1c between the placebo and either vitamin D_2_ or vitamin D_3_ groups with 90% power and a 5% significance level, assuming a standard deviation (s.d.) of HbA1c of 5.47 mmol/mol (0.5%). To detect a 5‐mmHg difference in mean systolic blood pressure between randomized groups, assuming an s.d. of 16 mmHg 15, yielded a requirement of 339 participants (113 in each group). Finally, to detect a 0.5‐m/s difference in PWV and assuming an s.d. of 1 m/s (S.E. Greenwald, unpublished observation), required 162 participants (54 in each group).

### Randomization and Blinding

Participants were randomized on a 1:1:1 basis within four strata defined by age (30–50 or 51–75 years) and sex, with a block size of six within each stratum. The order of treatments within each block was determined by a computer‐generated pseudo‐random sequence, generated by the IMP manufacturer (Nova Laboratories, Leicester, UK). Neither the participants, investigators, nor the laboratory staff knew the treatment allocation.

### Statistical Analysis

The primary analysis of efficacy outcomes used an intention‐to‐treat population, which included all participants in the group to which they were randomized, regardless of the treatment actually received. A secondary analysis used a per‐protocol population, which excluded individuals who did not take all doses of the IMP. The analysis of safety endpoints used a safety population, which included all participants in the group based on treatment actually received, thus it was identical to the intention‐to‐treat population.

The baseline characteristics of the study population were summarized separately within each randomized group using means and s.d. values (continuous variables), medians and interquartile ranges (skewed variables), or numbers and percentages (categorical variables). Change in HbA1c from baseline to 4 months was compared separately between each treatment group (D_2_ and D_3_) and placebo, using analysis of covariance (ancova), with adjustment for baseline and centre. To ensure that participants with missing baseline values for the outcome could be included in the analysis, the missing indicator method was used 20. An analysis was performed to check whether adjusting for age and sex (the randomization stratifiers) in the ancova model had any impact on the estimated treatment effects, with an *a priori* agreement not to include them in the model if the impact was minimal.

For each of the secondary efficacy outcomes, differences between each treatment group and placebo, together with 95% confidence intervals (CIs), were estimated using the same method as that described for the primary outcome. Continuous outcomes with skewed distributions [aspartate aminotransferase (AST), hsCRP] were natural log transformed. To enable the treatment differences for all efficacy outcomes to be reported on the same scale, each estimated difference and CI was divided by the s.d. of the relevant outcome at baseline. The number and percentage of participants experiencing any safety endpoints were reported within each randomized group. For the primary endpoint, prespecified interactions between treatment group and baseline HbA1c, and treatment group and baseline 25(OH)D were tested by including multiplicative interaction terms in the ancova model. For the primary efficacy endpoint only, an exploratory analysis was performed in which a difference between the D_2_ and D_3_ groups was estimated using ancova, as described above. All analyses were performed using stata 13 (Statacorp, College Station, TX, USA).

## Results

**Figure 1:**
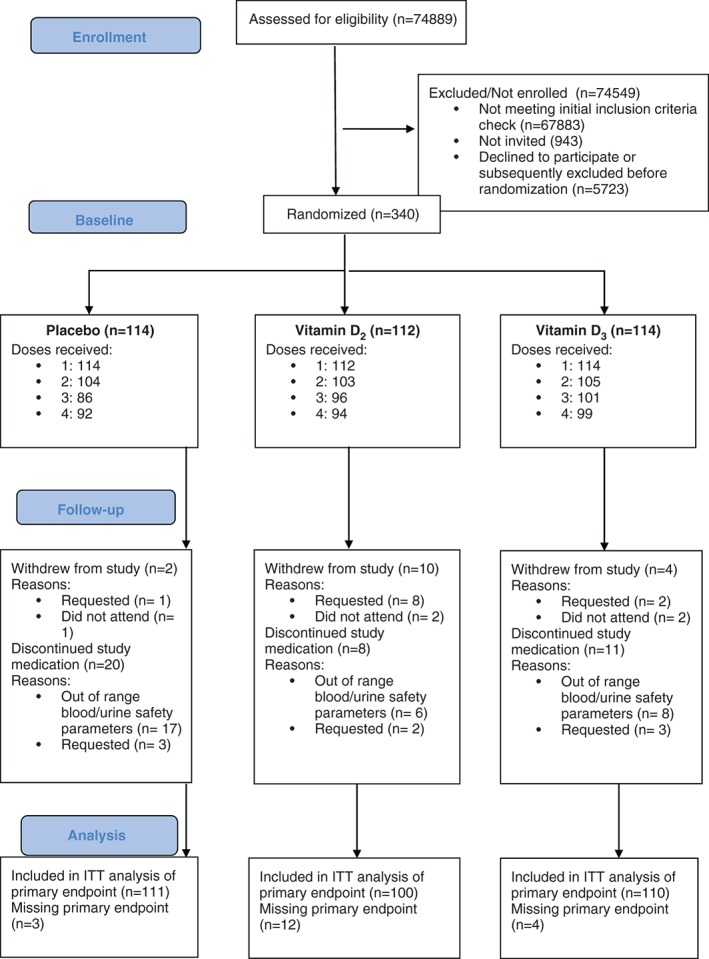
Flow diagram of participant recruitment into the vitamin D supplementation trial. ITT, intention to treat.

The recruitment of participants for the trial and their follow‐up occurred between 2010 and 2012 and was continuous throughout all seasons over the recruitment period. Of 340 participants, 114 were randomized to placebo, 112 to D_2_ and 114 to D_3_ (Figure 1). The distribution of baseline demographic, clinical and biochemical characteristics was similar across the treatment groups (Table 1). The percentages of participants who took all four doses of their randomized medication were 80.7% (placebo), 83.9% (D_2_) and 86.8% (D_3_; Figure 1).

**Table 1:** Baseline demographic and clinical characteristics of participants by randomized group.

		Placebo (N = 114)	**D_2_ (N = 112)**	**D_3_ (N = 114)**
	Total number of missing values	**Mean (s.d.) or median (IQR)**	**Mean (s.d.) or median (IQR)**	**Mean (s.d.) or median (IQR)**
Age, years	1	52.4 (8.5)	53.5 (8.7)	52.5 (8.2)
Body mass index, kg/m^2^	2	28.3 (5.0)	28.9 (5.5)	29.0 (5.5)
Systolic blood pressure, mm	0	127.9 (16.4)	126.9 (17.8)	128.6 (14.3)
Diastolic blood pressure, mm	0	77.6 (10.6)	75.8 (10.9)	77.6 (8.8)
Total 25(OH)D, nmol/l	1	51.1 (26.7)	53.8 (24.4)	51.2 (22.1)
25(OH)D_2_, nmol/l	1	5.2 (3.3)	5.2 (4.1)	5.4 (4.8)
25(OH)D_3_, nmol/l	1	45.8 (26.3)	48.6 (24.7)	45.8 (22.6)
HbA1c, %	2	5.9 (0.4)	5.9 (0.4)	5.9 (0.3)
HbA1c, mmol/mol	2	40.9 (3.8)	40.9 (4.2)	40.7 (3.4)
Total cholesterol, mmol/l	1	5.2 (1.1)	5.1 (0.9)	5.2 (0.9)
HDL cholesterol, mmol/l	1	1.3 (0.3)	1.3 (0.3)	1.3 (0.3)
Total/HDL ratio	1	4.3 (1.3)	4.0 (1.0)	4.2 (1.1)
Apolipoprotein A1, mmol/l	1	1.4 (0.3)	1.5 (0.2)	1.5 (0.3)
Apolipoprotein B, mmol/l	1	0.9 (0.2)	0.9 (0.2)	1.0 (0.2)
Modelled CVD risk, %	13	12.8 (9.4)	11.8 (8.3)	12.1 (8.2)
C‐reactive protein, mg/l	1	1.5 (0.7, 3.2)	1.4 (0.7, 3.3)	2.0 (0.8, 4.3)
Fructosamine, µmol/l	1	236.2 (20.9)	240.1 (24.3)	237.2 (21.9)
Parathyroid hormone, pmol/l	2	5.1 (2.5)	5.2 (2.0)	5.3 (2.2)
Alkaline phosphatase, IU/l	1	72.7 (19.3)	74.3 (19.4)	74.1 (19.4)
Aspartate aminotransferase, IU/l	2	19.0 (17.0, 24.0)	19.0 (16.0, 22.0)	20.0 (17.0, 23.0)
Pulse wave velocity*, m/s	8	7.4 (2.0)	7.3 (2.7)	7.9 (2.0)

		**N (%)**	**N (%)**	**N (%)**
*Centre*				
Cambridge		58 (50.9)	56 (50.0)	58 (50.9)
London		56 (49.1)	56 (50.0)	56 (49.1)
*Sex*	0			
Men		66 (57.9)	63 (56.3)	65 (57.0)
Women		48 (42.1)	49 (43.8)	49 (43.0)
*Smoking*	5			
Never		49 (43.0)	54 (48.2)	51 (44.7)
Former		35 (30.7)	35 (31.3)	36 (31.6)
Current		28 (24.6)	21 (18.8)	26 (22.8)
*Ethnic group*	2			
White		85 (74.6)	89 (79.5)	95 (83.3)
Non‐white		27 (23.7)	23 (20.5)	19(16.7)

25(OH)D, serum 25‐hydroxyvitamin D; CVD, cardiovascular disease; HbA1c, glycated haemoglobin.

* Pulse wave velocity was only measured at one centre (London), and includes 52, 55 and 53 participants in each treatment group, respectively.

**Figure 2:**
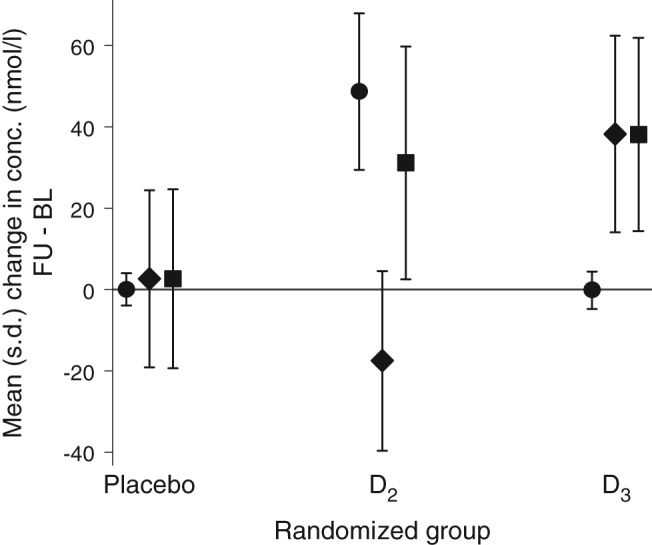
Mean [standard deviation (s.d.)] change in serum 25‐hydroxyvitamin D [25(OH)D]_2_, 25(OH)D_3_ and total 25(OH)D concentration (nmol/l) from baseline (BL) to 4 months follow‐up (FU), by randomized group. Circles = 25(OH)D_2_; diamonds = 25(OH)D_3_; squares = 25(OH)D.

The percentages of individuals with a 25(OH)D concentration <50 nmol/l at baseline were 58.8, 50.9 and 50.9% in the placebo, D_2_ and D_3_ groups, respectively; at follow‐up these percentages were 47.3, 4.5 and 3.5%. Mean (s.d.) 25(OH)D_2_ concentrations increased in the D_2_ group from 5.2 (4.1) nmol/l to 53.9 (18.5) nmol/l, and mean (s.d.) 25(OH)D_3_ concentrations increased from 45.8 (22.6) nmol/l to 83.8 (22.7) nmol/l in the D_3_ group. Between baseline and 4 months there was no overall change in concentrations of 25(OH)D_2_, 25(OH)D_3_ and total 25(OH)D in the placebo group (Figure 2). In contrast, in the D_2_ group, the mean 25(OH)D_2_ concentration increased by 48.7 (19.2) nmol/l, while the 25(OH)D_3_ concentration decreased by 17.5 (22.1) nmol/l, and total 25(OH)D concentration increased by 31.2 (28.6) nmol/l. In the D_3_ group there was no change in mean 25(OH)D_2_ concentration, while 25(OH)D_3_ and total 25(OH)D concentrations increased by 38.3 (24.2) nmol/l and 38.1 (23.8) nmol/l, respectively.

**Figure 3:**
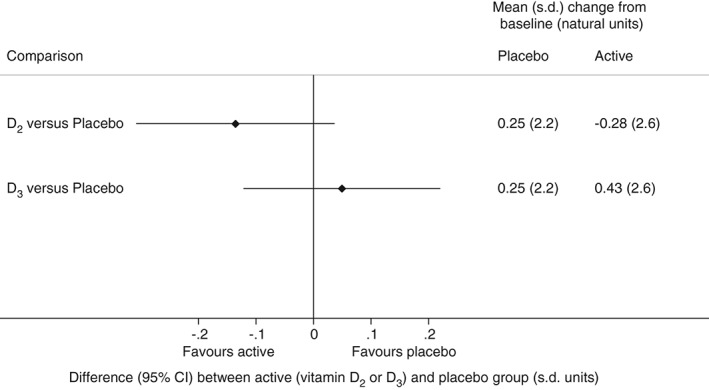
Difference [95% confidence interval (CI)] in the primary outcome (HbA1c) between vitamin D_2_ and placebo and between vitamin D_3_ and placebo groups, reported in units of baseline standard deviation (s.d.; 3.8 mmol/mol). Mean (s.d.) change from baseline is presented in each group in the natural units of the outcome (mmol/mol).

**Figure 4:**
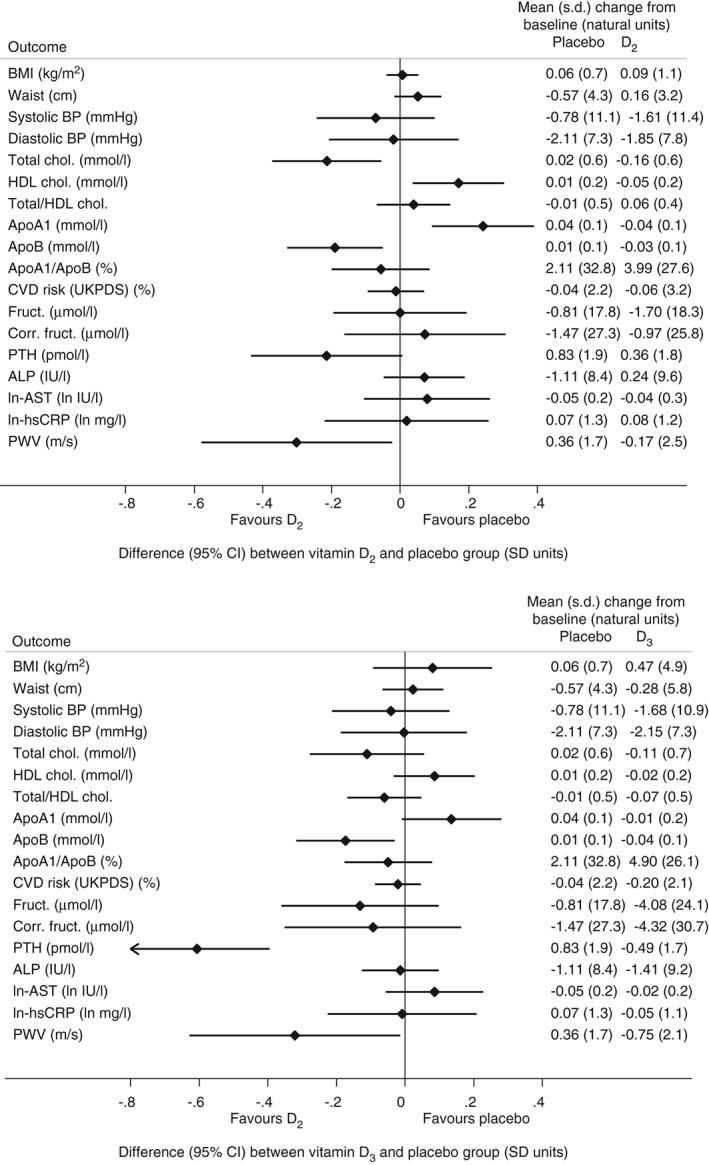
Difference [95% confidence interval (CI)] in secondary outcomes between D_2_ (A) and D_3_ (B) and placebo group, reported in units of baseline standard deviation (s.d.), alongside mean (s.d.) change from baseline in each group in the natural units of the outcome. BMI, body mass index; BP, blood pressure; chol., cholesterol; ApoA1, apolipoprotein A1; ApoB, apolipoprotein B; fruct., fructoasmine; corr. fruct., corrected fructosamine; ALP, alkaline phosphatase; AST, aspartame transaminase; CVD, cardiovascular disease; UKPDS, United Kingdom Prospective Diabetes Study; hsCRP, high‐sensitivity C‐reactive protein; PTH, parathyroid hormone; PWV, pulse wave velocity.

Figure 3 shows that there was no evidence of a difference between the placebo and vitamin D groups for the primary outcome, HbA1c: the difference comparing D_2_ with placebo was −0.05% (95% CI −0.11, 0.02) or −0.51 (95% CI −1.16, 0.14) mmol/mol (p = 0.13), and for D_3_ versus placebo it was 0.02% (95% CI −0.04%, 0.08%) or 0.19 (95% CI −0.46, 0.83) mmol/mol (p = 0.57). Among the secondary outcomes, there were no differences comparing D_2_ with placebo (Figure 4A) or D_3_ with placebo (Figure 4B) for anthropometric measures, blood pressure, hsCRP, CVD risk, assessed by UKPDS risk engine, hepatic markers, or fructosamine. In the D_2_ group there were small, but statistically significant, decreases from baseline relative to placebo in total cholesterol and ApoB (favouring D2), as well as in HDL cholesterol and ApoA1 (favouring placebo; Figure 4A). In the D_3_ group, there was a very small decrease in ApoB concentration between baseline and follow‐up relative to placebo (Figure 4B). There was an increase in PTH in the D_2_ group, but the difference between D_2_ and placebo was not statistically significant (Figure 4A). There was a decrease in PTH in the D_3_ group between baseline and follow‐up that was significantly different from the increase seen in the placebo group (Figure 4B). There was a reduction from baseline in PWV in both the D_2_ [mean (s.d.) −0.17 (2.5) m/s] and D_3_ [mean (s.d.) −0.75 (2.1) m/s] groups, relative to placebo [treatment difference D_2_ vs placebo: −0.68 (95% CI −1.31,−0.05) m/s; D_3_ vs placebo: −0.73 (95% CI −1.42, −0.03) m/s; Figure 4A, B]. In the per‐protocol population the findings were similar overall, but of greater magnitude, in particular for PWV, for which the treatment differences were −0.79 (95% CI −1.43, −0.14) m/s for D_2_ versus placebo, and −1.01 (95% CI −1.69, −0.34) m/s for D_3_ versus placebo.

In the prespecified analyses, there was no evidence for interaction between treatment group and either baseline HbA1c or baseline total 25(OH)D concentration on the primary outcome (all p values between 0.15 and 0.74). In *post hoc* analyses there was also no evidence of interaction between treatment group and ethnicity (white vs. non‐white ethnicity) or baseline 25(OH)D_3_ concentration (<50 vs. ≥50 nmol/l). In the prespecified exploratory analysis comparing HbA1c between D_3_ and D_2_ groups, there was no evidence of a treatment effect [D_3_ vs D_2_: 0.06% (95%CI −0.005, 0.13%) or 0.66 (95% CI −0.05, 1.38) mmol/mol]. In a *post hoc* analysis there was also no evidence of a difference in the change in fructosamine; D_3_ versus D_2_: −2.94 (95% CI −8.37, 2.48) µmol/l. There were no important safety issues related to vitamin D supplementation (Table S1).

## Discussion

Among individuals who are at elevated risk of future diabetes, monthly supplementation for 4 months with vitamin D_2_ and vitamin D_3_ at a dose equivalent to 3300 IU/day was efficacious in raising concentrations of 25(OH)D_2_ and 25(OH)D_3,_ respectively, but there were no differences between the placebo and either of the vitamin D supplementation groups for the primary outcome of change in HbA1c concentration._._ The null effects of vitamin D supplementation on HbA1c, blood pressure and inflammation in the present study suggest that vitamin D supplementation is unlikely to have major benefit for diabetes prevention or cardiometabolic risk. Nevertheless, the reduction we observed in arterial stiffness, as assessed by PWV, is of interest. The trial also showed the feasibility of relatively high dose supplementation over 4 months in a population unscreened for baseline 25(OH)D concentrations.

The null findings for change in HbA1c are in keeping with evidence from clinical trials appraised in two previous systematic reviews and meta‐analyses 2, 3. Evidence from vitamin D supplementation trials conducted subsequently to these meta‐analyses is also supportive of no significant effect on HbA1c concentrations 4, 10, 21. One trial did report a net benefit, with 0.2% lower HbA1c concentration in the vitamin D‐supplemented group (n = 56) versus placebo (n = 53), but this trial differed from other published work substantially, with very‐high‐dose vitamin D_3_ supplementation (a mean dose of ∼88 000 IU/week, equivalent to >12 000 IU/day), longer duration of the intervention (for 1 year), and participants restricted to those of Latino or African‐American ethnicity, together with presence of both impaired glucose regulation [HbA1c of 5.8–6.9% (40–52 mmol/mol)], plus impaired fasting glucose or impaired glucose tolerance, and hypovitaminosis‐D defined as 25(OH)D concentrations <75 nmol/l 5. Notably, no other marker of glycaemia or insulin resistance differed by treatment group in that trial.

The null effect in the present study of supplementation on several prespecified secondary outcomes is unsurprising in light of the previous similar null reports for anthropometric markers 8, 21 and markers of cardiometabolic risk, including blood pressure 8, 10, 22, 23 C‐reactive protein 8, 9, 10, 24, 25 and liver enzymes 26, while our null finding for the UKPDS CVD risk engine risk score is novel. Our observation of minor effects on some lipid and Apo values is congruent with the past mixed evidence for effects of vitamin D supplementation on lipids 10, 27. A recent trial reported significantly reduced concentrations of ApoB in the vitamin D‐supplemented group versus placebo, but similarly to our findings, the small magnitude of change was not considered clinically significant 28, while an ‘umbrella’ review of meta‐analyses showed generally null findings for lipid outcomes 29.

Our finding of a decrease in PWV between follow‐up and baseline in both the D_2_ and D_3_ groups relative to placebo is at variance with the null findings for carotid‐femoral PWV previously reported from a trial in 100 patients with diabetes, where supplementation for 12 weeks with 5000 IU/day of vitamin D_3_ versus placebo did not have an effect on brachial‐ankle PWV 12. Other trials have reported conflicting findings in people without diabetes 11, 30, 31, 32, 33. Mechanisms underlying the reduction in arterial stiffness by supplementation with both vitamin D_2_ and D_3_ are unestablished, but possible pathways include both direct and indirect effects on vascular cells, suppression of the renin‐angiotensin‐aldosterone system, effects on calcium metabolism leading to the calcification of arterial elastin and the interplay of inflammation and oxidative stress 23, 31, 34, 35. Our findings suggest that supplementation with either vitamin D_2_ or D_3_ over a 4‐month period could offer a way to potentially favourably affect arterial stiffness, perhaps by inhibition of matrix metalloproteinases 36, 37, and should stimulate further research to replicate these findings and to understand the mechanisms of how 25(OH)D concentrations may exert functional and structural alterations in the arterial system.

While overall the total 25(OH)D concentrations were increased in both D_2_ and D_3_ groups in the present trial, the magnitude of increase in 25(OH)D was greater with D_3_ supplementation (mean increase of 38.3 nmol/) than with D_2_ supplementation (mean increase of 31.2 nmol/l), in keeping with a previous meta‐analysis comparing the effects of supplementation with D_2_ or D_3_ 13 and with trials conducted since then 14, 38. This may be partially attributable to the shorter half‐life of 25(OH)D_2_ versus 25(OH)D_3_ in the circulation, which is related to the lower affinity of the D_2_ metabolite for the vitamin D‐binding protein 39. It might also reflect our finding that supplementation with vitamin D_2_ led to a decrease in 25(OH)D_3_ concentrations, as also observed in non‐trial settings by others 40, 41, while supplementation with vitamin D_3_ did not affect 25(OH)D_2_ concentrations in the present trial. The decrease in 25(OH)D_3_ concentrations in the D_2_ group may suggest different bioavailability 38, possible more rapid metabolism or clearance of circulating 25(OH)D_3_ following D_2_ supplementation, or competition for enzymatic activity by CYP2R1 for 25‐hydroxylation of vitamin D_2_ and D_3_ 41. It has been suggested that an upregulation in mechanisms required to metabolize D_2_ and its metabolites may increase the degradation of circulating 25(OH)D_3_ concentrations 42 but the biological significance of these changes is currently unclear.

The strengths of the present trial include the cohort of adults from different ethnic groups, of varying ages and both sexes, and the use of a relatively high dose of vitamin D supplementation at the daily equivalent dose of 3300 IU per day that was effective in raising 25(OH)D concentrations. The comparison of both vitamin D_3_ and vitamin D_2_ against placebo for cardiometabolic outcomes is novel. Our inclusion of several relevant secondary endpoints and the assessment of the feasibility and safety of 25(OH)D in relatively high doses given monthly in a general population unscreened for 25(OH)D concentrations, with high degree of participant retention, increases generalizability in clinical practice. Enrolling participants irrespective of baseline vitamin D status was intentional to enable feasibility of a ‘real‐life’ trial that would not require pre‐recruitment assessment, and our examination of any differential effects by baseline 25(OH)D concentration in interaction analyses highlighted no benefits in those with hypovitaminosis D.

The limitations of the present study included the inability to draw conclusions for longer‐term effects of supplementation beyond 4 months. For the primary glycaemic endpoint of HbA1c, this period could be considered short, but our null findings for this outcome were supported by similar null findings for fructosamine concentrations, yet we observed bioactivity for PWV, lipid parameters and PTH. The study did not allow us to compare D_2_ with D_3_ supplementation directly because of sample size constraints, but an exploratory analysis was null. The PWV findings are interesting but we acknowledge the potential limitations of using surrogate markers of CVD. Finally, the present study did not address whether different outcomes would have been obtained with a daily compared with a monthly regime.

In summary, short‐term supplementation with vitamin D_2_ and D_3_ raised concentrations of 25(OH)D_2_ and 25(OH)D_3_, respectively, but had no effect on HbA1c during the study period. The modest reduction in PWV with both D_2_ and D_3_ versus placebo is suggestive of a beneficial effect of vitamin D supplementation on arterial stiffness, and the clinical implications of this finding merit further investigation.

## Conflict of Interest

None of the authors has any conflict of interest.

All authors participated in the design of the study. S. J. S. wrote the statistical analysis plan and conducted statistical analyses. N. G. F. and G. A. H. drafted the manuscript. G. A. H. was chief investigator and N. G. F. was the lead investigator. G. A. H., S. J. G., N. G. F., R. M., N. M. and A. P. R. were responsible for the conduct and monitoring of the trial. N. G. F. and G. A. H. are guarantors and take responsibility for the contents of the article. P. M. T. advised on and conducted laboratory measurements. A. M., B. J. B., T. A. C., C. J. G. and S. E. G. advised on trial related issues. R. M. and N. M. conducted data collection in London. All authors provided intellectual input and read and approved the final version of the manuscript.

## Supporting information


**Table S1.** Number (%) of individuals with safety endpoints, by randomised group‐vitamin D supplementation trial.
