# Supplementary material for: Effects of vitamin D2 or D3 supplementation on glycaemic control and cardiometabolic risk among people at risk of type 2 diabetes: results of a randomized double‐blind placebo‐controlled trial
Source: Diabetes Obes Metab. 2016 Feb 4;18(4):392–400. doi: 10.1111/dom.12625 (PMC4950066; doi:10.1111/dom.12625)
Supplement: Supplementary file 1 — Table S1. Number (%) of individuals with safety endpoints, by randomised group‐vitamin D supplementation trial. [file DOM-18-392-s001.docx]

Supplementary Table S1: Number (%) of individuals with safety endpoints, by randomised group- vitamin D supplementation trial.

|  | **Placebo** | | **D_2_** | | **D_3_** | |
| --- | --- | --- | --- | --- | --- | --- |
|  | **(N=114)** | | **(N=112)** | | **(N=114)** | |
|  | % | N | % | N | % | N |
|  |  |  |  |  |  |  |
| Any adverse event (AE) | 62.3 | 71 | 68.8 | 77 | 59.6 | 68 |
|  |  |  |  |  |  |  |
| Any serious adverse event (SAE) | 0.0 | 0 | 1.8 | 2 | 3.5 | 4 |
|  |  |  |  |  |  |  |
| Any adverse reaction (AR) | 0.9 | 1 | 0.0 | 0 | 0.0 | 0 |
|  |  |  |  |  |  |  |
| Any suspected serious adverse reaction (SSAR) | 0.0 | 0 | 0.0 | 0 | 0.0 | 0 |
|  |  |  |  |  |  |  |
| Any suspected unexpected serious adverse reaction (SUSAR) | 0.0 | 0 | 0.0 | 0 | 0.0 | 0 |
|  |  |  |  |  |  |  |
| Ionised calcium>1.3 mmol/l | 3.5 | 4 | 4.5 | 5 | 5.3 | 6 |
|  |  |  |  |  |  |  |
| Serum corrected calcium > 2.65 mmol/l | 0.0 | 0 | 0.0 | 0 | 0.0 | 0 |
|  |  |  |  |  |  |  |
| Ionised calcium>1.3 mmol/l or serum corrected calcium > 2.65 mmol/l | 3.5 | 4 | 4.5 | 5 | 5.3 | 6 |
|  |  |  |  |  |  |  |
| Urine calcium:creatinine ratio > 1 | 7.0 | 8 | 5.4 | 6 | 5.3 | 6 |
|  |  |  |  |  |  |  |
